# Revision quadriceps tendon repair: A case series and technique guide to a novel repair

**DOI:** 10.1016/j.tcr.2025.101132

**Published:** 2025-01-04

**Authors:** Ellen Lutnick, Sophia Puertas, Mark Anders

**Affiliations:** Department of Orthopaedic Surgery and Sports Medicine, University at Buffalo, Jacobs School of Medicine and Biomedical Sciences, Buffalo, NY 14203, United States of America

**Keywords:** Quadriceps repair, Extensor mechanism disruption, Revision quadriceps repair

## Abstract

**Introduction:**

Revision quadriceps tendon repair is a challenging problem. In this four-case series, novel quadriceps tendon revision resulted in improved range of motion and durable repair for patients with recurrent rupture.

**Methods:**

Our technique includes a combination of a running locked #5 FiberWire or 2 mm SutureTape suture placed through parallel medial, lateral, and central drill holes in the patella with running Krackow-type quadriceps tendon repair medially and laterally resulting in four strands, delivering the vastus medialis and medial quadriceps tendon to an anatomic repair at the superior pole of the patella, with 2 sutures passed centrally and 1 each passed medially and laterally and then tied. Reinforcement is performed using a tibialis anterior tendon allograft with placement at the inferior pole of the patella starting superolaterally coursing lateral to medial through infrapatellar tendon. It is then threaded medially into the centrally repaired portion of the quadriceps tendon, and then back down to the lateral suprapatellar and lateral patellar retinaculum, giving three crossing strands. This is repaired with multiple interrupted 0 Vicryl mattress sutures. Immobilization postoperatively was dictated by patient's body habitus.

**Results:**

Patient 1 was a 79-year-old obese man treated after two prior revision periprosthetic quadriceps repair procedures. He was immobilized in a knee immobilizer for 8 weeks postoperatively. He was revised for TKA instability at 6 months postoperatively, and one month later returned to the operating room for persistent hematoma; repair was noted to be intact. Patient 2 was a 39-year-old morbidly obese man who was revised after failure of one revision quadriceps repair. He was protected with an external fixator for 6 weeks. Patient 3 was a 49-year-old obese man who was treated with four revision quadriceps repair procedures over the course of 15 years. Postoperatively he was treated with a knee immobilizer. Patient 4 was a 71-year-old obese man who was treated after failure of one prior revision quadriceps repair procedure. He was casted postoperatively for one month. On final follow up, all patients were able to maintain straight leg raise, with functional range of motion and ambulation.

**Conclusion:**

Revision quadriceps tendon repair using an anterior tibialis tendon allograft is a viable solution for obese patients with recurrent quadriceps tendon ruptures.

## Introduction

Quadriceps femoris tendon rupture is an uncommon injury typically occurring in patients over 40 years old in the setting of systemic disease [1,2] Partial quadriceps tears can often be managed nonoperatively through immobilizer usage and physical therapy, but complete tears are disabling and standard of care includes surgical treatment as soon as possible [3].

First line surgical management typically includes the placement of suture loops through transpatellar tunnels [4]. The use of suture anchors in place of transpatellar tunnels has been more recently introduced in the literature [5]. However, regardless of technique, significant challenges such as pain and stiffness, inability to return to work or sport, persistent quadriceps weakness and re-rupture have been reported to with current quadriceps repair techniques [6,7]. With failure of primary quadriceps repair estimated consistently around 5% across the literature [8], successful revision quadriceps repair methods are essential.

Revision quadriceps repair procedures often face challenges such as scar tissue buildup and tendon retraction, leading to complicated specialized surgeries typically still with poor functional outcomes [9]. A few novel revision quadriceps repair techniques have been previously published; most of these techniques have focused on using tendon auto or allografts and modifications or combinations of common quadriceps repair techniques. Most relevant to the technique proposed in the study at hand are the auto/allograft techniques, such as an ipsilateral hamstring autograft [10], Achilles tendon allograft [11], and patella-quadriceps tendon allograft [12]. However, due to the rarity of the injury, sample sizes can be very small and there is a paucity of literature related to revision quadriceps repair.

We present the cases of four individuals with recurrent quadriceps tendon tears who underwent multiple revision surgeries. In the lattermost revision for each, a tibialis anterior tendon allograft was used, and this is the first known report of its usage in revision quadriceps repair. This novel revision quadriceps repair technique has shown to result in improved patient functional outcomes in our patient cohort.

The patients were informed that this case would be submitted for publication and agreed to the submission of associated data.

## Methods

**Fig. 1:**
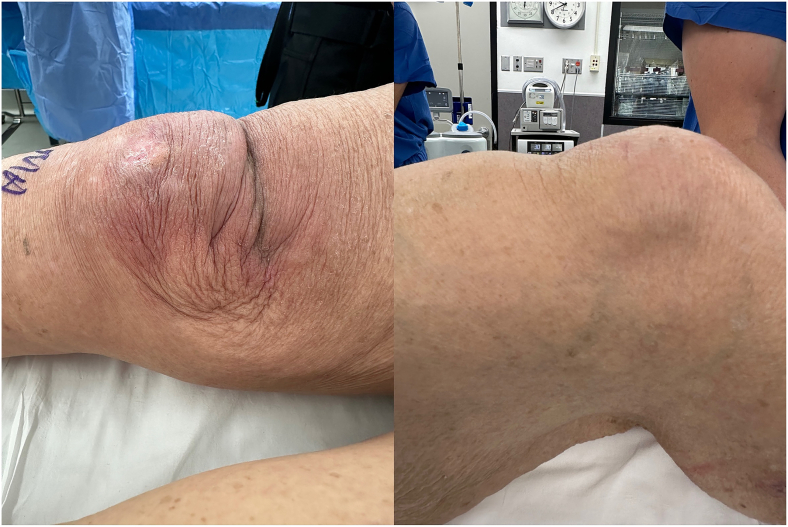
Clinical example of a patient with quadriceps tendon re-rupture, with palpable and visible quadriceps tendon defect.

**Fig. 2:**
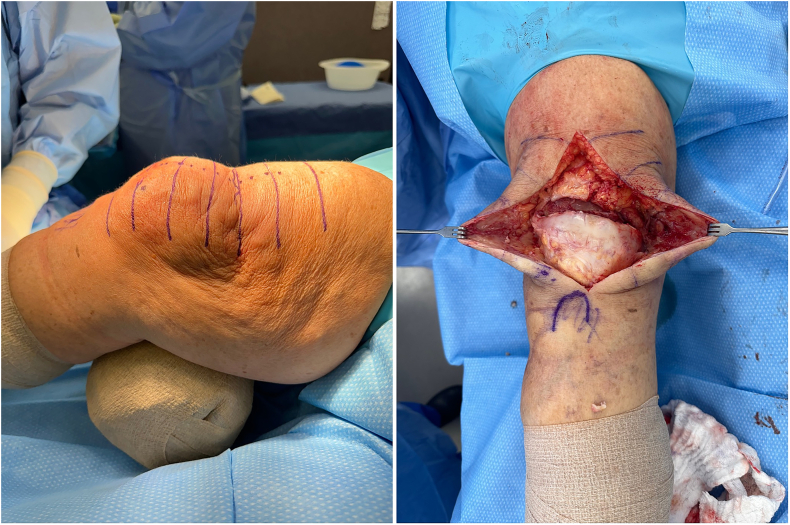
The quadriceps defect is accessed through a standard midline approach.

**Fig. 3:**
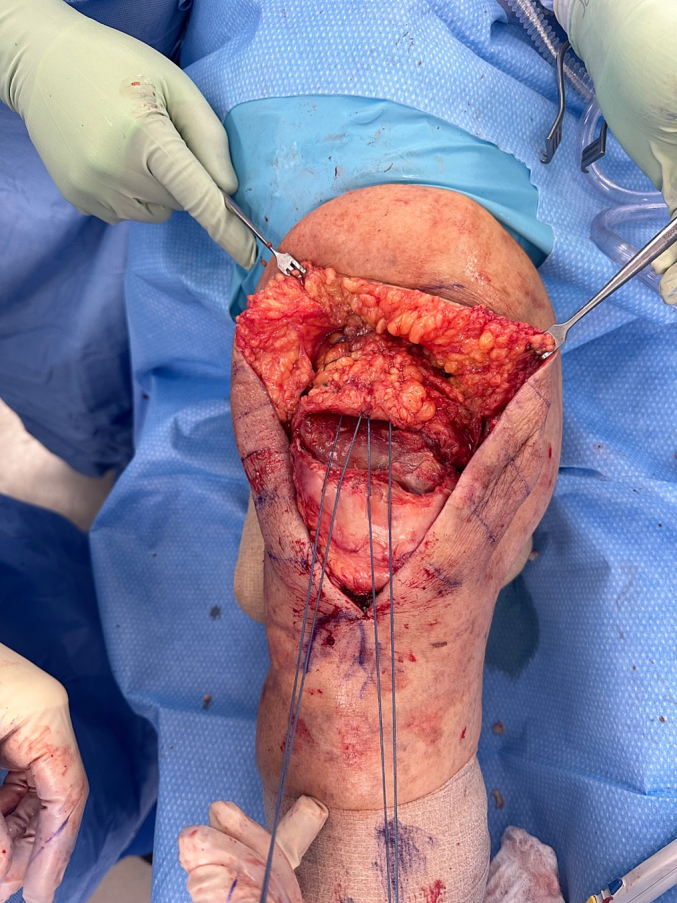
A running locked #5 FiberWire or 2 mm SutureTape suture in typical Krackow-style fashion is repaired medially and laterally resulting in four strands, delivering the vastus medialis and medial quadriceps tendon to an anatomic repair at the superior pole of the patella.

**Fig. 4:**
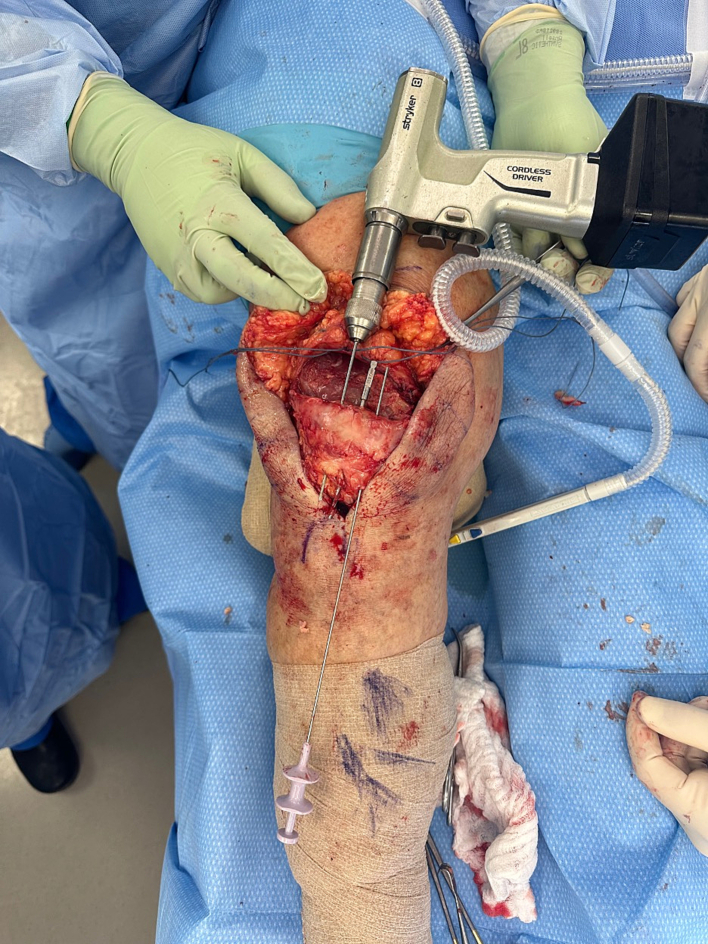
The patella is prepared with parallel medial, lateral, and central drill holes.

**Fig. 5:**
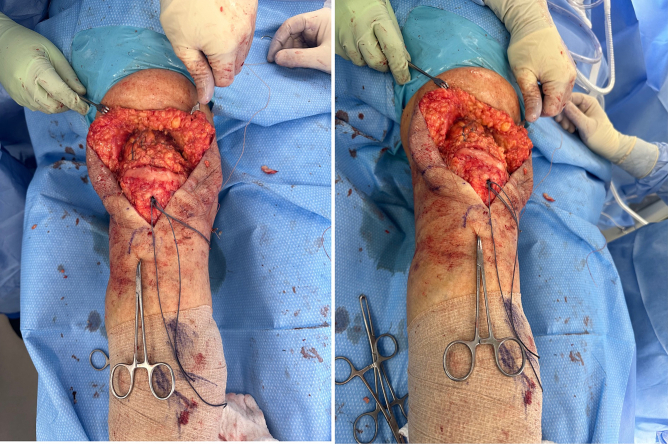
Once patella drill holes are prepared, two of the quadriceps tendon sutures are passed centrally, and 1 each passed medially and laterally and then tied.

A novel allograft repair method was performed in patients whose primary repair procedures had resulted in re-rupture. Fig. 1 demonstrates one clinical example of a patient with quadriceps tendon re-rupture, with palpable and visible quadriceps tendon defect. The quadriceps defect is accessed through a standard midline approach (Fig. 2). Once the defect is fully visualized and cleaned, our technique includes a combination of a running locked #5 FiberWire or 2 mm SutureTape suture in typical fashion with running Krackow-type repair medially and laterally resulting in four strands, delivering the vastus medialis and medial quadriceps tendon to an anatomic repair at the superior pole of the patella (Fig. 3). The patella is then prepared with parallel medial, lateral, and central drill holes (Fig. 4), and two of the quadriceps tendon suture are passed centrally, and 1 each passed medially and laterally and then tied. Any injury to the surrounding paratenon medial and lateral to the patella are reinforced with additional running locking #2 Vicryl suture (Fig. 5).

**Fig. 6:**
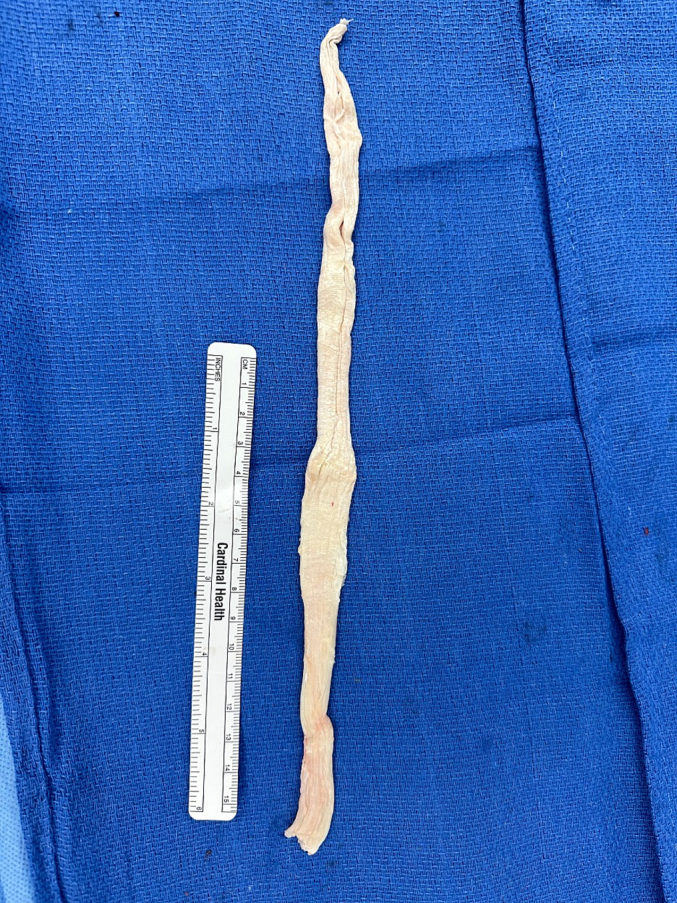
Ideally, the tibialis anterior tendon allograft is >25 cm to supplement the repair.

**Fig. 7:**
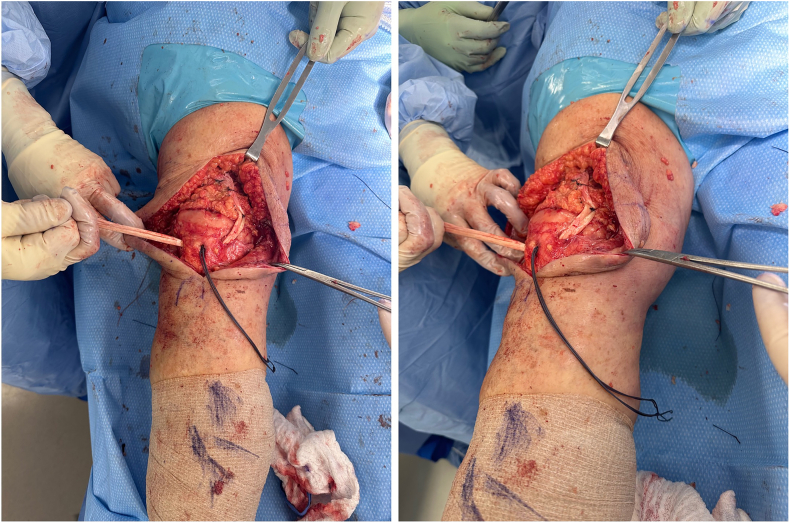
Allograft woven across the superior patella and quadriceps: first, placed at the inferior pole of the patella through infrapatellar tendon, then threaded back up medially into the centrally repaired portion of the quadriceps tendon, and then back down to the lateral suprapatellar and lateral patellar retinaculum.

**Fig. 8:**
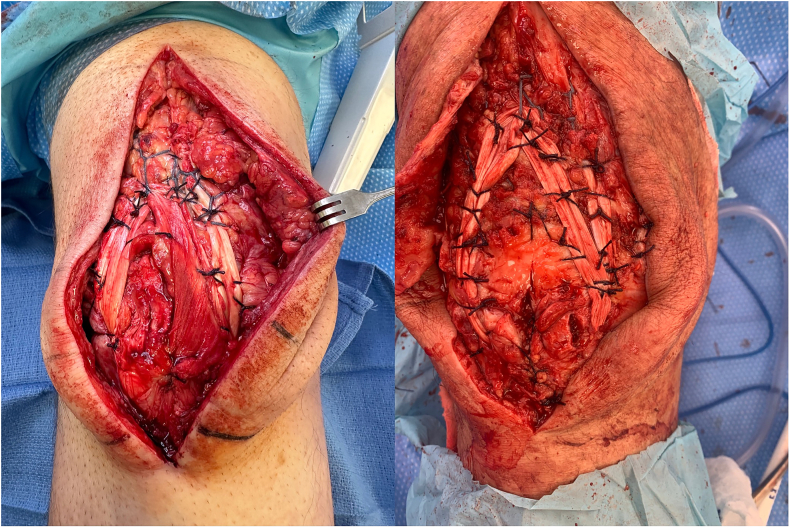
Finalized repair demonstrate anatomic reduction of the quadriceps tendon to the superior pole of the patella, with allograft repair in place.

Reinforcement of this repair is performed using a tibialis anterior tendon allograft (ideally >28 cm, Fig. 6), which supplements the repair. This allograft allows for 3 limbs crossing the superior patella and quadriceps with placement at the inferior pole of the patella starting superolaterally coursing lateral to medial through infrapatellar tendon. It is then threaded back up medially into the centrally repaired portion of the quadriceps tendon, and then back down to the lateral suprapatellar and lateral patellar retinaculum (Fig. 7). The allograft is then repaired with multiple interrupted 0 Vicryl mattress sutures. The primary suture knot is buried in the patellar tendon and medial retinaculum, reinforced with several #2 Vicryl sutures. Fig. 8 demonstrates several examples of this final repair. Immobilization after this revision includes bracing, casting, or external fixation depending on the patient's body habitus.

## Cases

### Case 1

Case 1 involves a 79-year-old obese man who had previously undergone two revision periprosthetic quadriceps repair procedures after total knee arthroplasty. Preoperatively, he was noted to have a palpable defect as well as extensor lag and reported an inability to navigate stairs. He was treated using our novel technique, and postoperatively immobilized in a knee immobilizer for 8 weeks. On follow up at 4 months he was able to fully flex and extend at the knee. At 6 months postoperatively he was revised for TKA instability using an upsized polyethylene insert. At this time, quadriceps repair was noted to be intact. At 7 months after revision he presented in the operating room for persistent hematoma. At final follow up 8 months after revision he was noted to have an intact extensor mechanism as well as be able to maintain a straight leg raise.

### Case 2

Case 2 involves a 39-year-old morbidly obese (BMI 62) man presented with primary repair failure due to a slip and fall. Preoperatively, he presented with loss of extensor function. After our novel surgical repair, an external fixator from the anterolateral femur to the tibia was utilized for 6 weeks due to the patient's body habitus and injury complexity. On final follow up at 5 months, the patient was able to demonstrate extensor resistance, maintain a straight leg raise in the dependent position, and flex to 90 degrees.

### Case 3

Case 3 involves a 49-year-old obese man who presented with a history of four revision quadriceps repair procedures over the course of 15 years. His most recent repair had failed by traumatic mechanism, and he reported a sensation of instability. After implementing our novel repair, his knee was placed in a bulky dressing and range of motion knee brace locked in extension. On final follow up at 4 months after revision, the patient was able to initiate and maintain a straight leg raise. He demonstrated an intact extensor mechanism and return of functional ambulation without knee immobilizer, and was scheduled for physical therapy to start quadriceps strengthening.

### Case 4

Case 4 is a 71-year-old obese man who was treated by our technique after failure of one prior revision quadriceps repair procedure. He presented preoperatively with a 30–40 degree extensor lag on straight leg raise. After surgical intervention with our novel repair, he was placed into a long leg cast for one month. After the cast was removed, the patient was able to maintain a straight leg raise without support and flex to approximately 30 degrees. On final follow up at 11 months, he demonstrated nearly symmetric quad strength clinically and was able range his knee from full extension and flexion of 110 degrees.

## Discussion

Quadriceps tendon ruptures are fairly rare, estimated about six times less frequent than patellar fractures [1,13]. Repair of quadriceps tendon ruptures is a technically challenging procedure with good result; however, re-rupture is a feared complication [14]. Obesity is both a risk factor for primary rupture, as well as a risk factor for failure of primary repair [15]. Several current methods of quadriceps revision have been previously published, although most are small studies with varying rates of success. Typically, like in our novel repair, these focus on allograft and autograft supplementation.

For example, an ipsilateral hamstring autograft technique has been published describing harvest of the gracilis and semitendinosus hamstrings from a separate 3 mm incision, preparation of the grafts by end-to-end repair to produce the graft utilized for reconstruction, and passage of the graft through a transverse tunnel through the midportion of the patella, and several times through the quadriceps tendon [10]. Additional augmentation with mesh overlying the repair, hamstring tendon, quadriceps and patella has also been described [16]. Our technique is novel in that it does not require a transverse drill hole through the patella for passage of the graft, which could possibly further weakening the bone.

An Achilles tendon allograft technique has been described, using a reamer to prepare a socket medial to the tibial tubercle, into which the calcaneal bone block of the Achilles allograft is fashioned to match and deep to the paratenon with an interference screw. Any remnant native quadriceps tendon is repaired using patella anchors with running Krakow sutures, and the soft tissue component of the Achilles graft is laid over the repair and oversewn with free nonabsorbable suture. In this study, all 14 patients could perform a straight leg raise, although 25% had a residual extensor lag; there were no incidence of failure of the repair at mean follow up of 52 weeks [11].

Another patella-quadriceps tendon allograft technique was detailed in one case report, describing use of allograft patella by removing the articular surface, and fixing the allograft to the anterior native patella with 3.5 mm and 2.7 mm unicortical screws. The quadriceps tendon on the allograft was then centrally repaired to the native quadriceps with the knee in full extension using multiple Bunnell sutures. Clinical outcomes included the patient with increased strength, less pain, 130 degrees of flexion and 20 degrees of extensor lag at 13 month follow up, as well as radiographic evidence of bridging bone between the native and allograft patella [12].

Our technique has demonstrated improvement in all four patients with follow-up times ranging from 3 months to 11 months. Improved active flexion and extension was noted as soon as 3–5 months postoperatively. Appropriate postoperative immobilization was also critical, informed by each individual patient's ability to maintain instructed postoperative weight bearing status, and body habitus. It is also important to note that all patient cases included in this series were obese or morbidly obese. Generally, obese patients often suffer from greater complications, longer recovery times, and poorer outcomes from quadriceps repair surgery [17]. Previous studies have found that obesity is associated with prolonged activation of the quadriceps muscle and that obese individuals often have lower strength in their quadriceps muscle, predisposing to initial injury and subsequent failure [18,19]. The correlation between obesity and quad muscle strength and activation must be considered in the trajectory of patient recovery and functional outcomes. Recovery results with this new revision technique have been favorable in all four patients despite their relative body habitus, which indicates a potentially durable repair option in this at-risk population.

The clinical outcomes of our novel technique are limited by the power of this case series and the length of follow up available for these patients. However, our highlighted technique shows promising results in the setting of a surgical challenge. As revision quadriceps repair using an anterior tibialis tendon allograft resulted in improved range of motion and durable repair in this four-case series, we suggest that the technique appears to be a viable solution for obese patients with recurrent quadriceps tendon ruptures. Based on encouraging results in these high-risk failures, this also may be a technique applicable for patients with other surgical challenges, including poor tissue quality and neglected initial injury leading to delayed treatment, which is currently being explored.

## Conclusion

We suggest that our novel technique of revision quadriceps repair presents one successful strategy for extensor mechanism restoration in the setting of a high-risk population, specifically in those obese patients with history of prior repair failure. This option, paired with appropriate postoperative immobilization, offers the possibility of functional restoration of the extensor mechanism with a reliable, cost-effective technique.

## CRediT authorship contribution statement

**Ellen Lutnick:** Data curation, Supervision, Writing – original draft, Writing – review & editing. **Sophia Puertas:** Data curation, Writing – original draft. **Mark Anders:** Conceptualization, Writing – review & editing.

## Funding source

No funding was requested for this project.

## Declaration of competing interest

The authors declare that they have no known competing financial interests or personal relationships that could have appeared to influence the work reported in this article.
